# Successful Treatment of T Cell-Mediated Acute Rejection with Delayed CTLA4-Ig in Mice

**DOI:** 10.3389/fimmu.2017.01169

**Published:** 2017-09-20

**Authors:** James S. Young, Stella H.-W. Khiew, Jinghui Yang, Augustin Vannier, Dengping Yin, Roger Sciammas, Maria-Luisa Alegre, Anita S. Chong

**Affiliations:** ^1^Department of Surgery, Section of Transplantation, The University of Chicago, Chicago, IL, United States; ^2^Department of Organ Transplantation, Shanghai Changzheng Hospital, Second Military Medical University, Shanghai, China; ^3^Center for Comparative Medicine, University of California, Davis, Davis, CA, United States; ^4^Department of Medicine, Section of Rheumatology, The University of Chicago, Chicago, IL, United States

**Keywords:** transplantation, allospecific, costimulatory blockade, CTLA4-Ig, T lymphocyte, heart, memory, sensitization

## Abstract

Clinical observations that kidney transplant recipients receiving belatacept who experienced T cell-mediated acute rejection can be successfully treated and subsequently maintained on belatacept-based immunosuppression suggest that belatacept is able to control memory T cells. We recently reported that treatment with CTLA4-Ig from day 6 posttransplantation successfully rescues allografts from acute rejection in a BALB/c to C57BL/6 heart transplant model, in part, by abolishing B cell germinal centers and reducing alloantibody titers. Here, we show that CTLA4-Ig is additionally able to inhibit established T cell responses independently of B cells. CTLA4-Ig inhibited the *in vivo* cytolytic activity of donor-specific CD8^+^ T cells, and the production of IFNγ by graft-infiltrating T cells. Delayed CTLA4-Ig treatment did not reduce the numbers of graft-infiltrating T cells nor prevented the accumulation of antigen-experienced donor-specific memory T cells in the spleen. Nevertheless, delayed CTLA4-Ig treatment successfully maintained long-term graft acceptance in the majority of recipients that had experienced a rejection crisis, and enabled the acceptance of secondary BALB/c heart grafts transplanted 30 days after the first transplantation. In summary, we conclude that delayed CTLA4-Ig treatment is able to partially halt ongoing T cell-mediated acute rejection. These findings extend the functional efficacy of CTLA4-Ig therapy to effector T cells and provide an explanation for why CTLA4-Ig-based immunosuppression in the clinic successfully maintains long-term graft survival after T cell-mediated rejection.

## Introduction

A high-affinity CTLA4-Ig, belatacept, was approved by the US Food and Drug Administration in 2011 for the prophylaxis of organ rejection in EBV-positive renal transplant recipients (1). The mechanism of action of belatacept relies on high-affinity binding to CD80 and CD86, thereby preventing CD28-mediated co-stimulation on T cells and their subsequent activation (2–5). A 7-year follow-up of the Belatacept Evaluation of Nephroprotection and Efficacy as First-Line Immunosuppression Trial (BENEFIT) confirmed the efficacy of belatacept; it reported that patient and graft survival, as well as mean estimated glomerular filtration rates (eGFR) were superior in patients receiving belatacept compared to patients receiving cyclosporine (CsA), despite higher incidence of early acute cellular rejection (6–10). Possible explanations include: CsA being an inferior immunosuppressant compared to tacrolimus, CsA’s nephrotoxicity, CsA-mediated reduction of mycophenolic acid exposure, and a significantly lower incidence of donor-specific antibody (DSA) development (1.9 and 4.6%) in the belatacept medium and low-intensity treatment groups, compared to 17.8% in the CsA-treatment cohort (10, 11). The link between DSA, especially anti-Class II antibodies, antibody-mediated rejection, and allograft failure has been well documented in multiple clinical studies (12–16), as patients have the propensity to develop new DSA after acute rejection (17). Thus, the observations of increased incidence of acute rejection but reduced DSA and superior outcome in patients treated with belatacept are intriguing and have prompted renewed investigations into the effect of CTLA4-Ig on established B cell/DSA and T cell responses, and for treating acute rejection (18–22).

In our previous studies, we tested the efficacy of delayed CTLA4-Ig treatments in controlling established alloreactive B cells and alloantibody responses in mice (18–21). Because non-adherence is more frequent in patients who developed DSA and progress to graft failure (15), we allowed alloreactive B and T cells responses to develop against fully MHC and minor mismatched allogeneic splenocyte immunization or heart transplantation for 6–7 days before initiating CTLA4-Ig treatment. Using a novel technique of tracking the fate of donor-specific B cells, we previously showed maximum numbers of donor-specific B cells with a germinal center (GC) phenotype by day 7–14 postimmunization, and the ability of CTLA4-Ig, starting on day 6–7 post-sensitization, to reduce the numbers of donor-specific GC B cells to baseline within 7 days and halt further increase in alloantibody titers (18–21). We also showed that treatment with CTLA4-Ig on day 6 post-heart transplantation inhibited DSA production and rescued fully MHC and minor mismatched heart grafts from acute rejection (21). Since heart allograft rejection in this BALB/c to C57BL/6 transplantation model is primarily mediated by T cells (23), we hypothesized that delayed CTLA4-Ig also modulated the function of effector T cells.

There is a considerable body of literature indicating that costimulation blockade, including CTLA4-Ig, fails to induce tolerance in sensitized mice (24–27); however, less is known of its effects on effector T cell function when blockade is maintained continuously. Clinical observations that acute rejection in belatacept-treated kidney transplant recipients is successfully rescued by transient steroid therapy and that grafts can be subsequently maintained on belatacept-based immunosuppression suggest that it may be effective at controlling established effector and, potentially, memory T cell function (8–10). In this study, we defined the effects of CTLA4-Ig on endogenous effector donor-reactive T cells and their function in a BALB/c to C57BL/6 heart transplantation model.

## Materials and Methods

### Mice

Female C57BL/6 (B6, H-2^b^), BALB/c (B/c, H-2^d^), and TCRβ^−/−^ mice (8–12 weeks old) were purchased from Harlan Laboratories (Madison, WI, USA), and μMT C57BL/6 were from Jackson Labs (Bar Harbor, ME, USA). 2W-OVA transgenic C57BL/6 mice (28) were bred with BALB/c mice to obtain 2W-OVA.F1 mice.

### Transplantation

Heterotopic heart transplantations were performed as previously described (21) by grafting BALB/c or 2W-OVA F1 hearts from 6- to 8-week-old donors onto the inferior vena cava and aorta in the peritoneal cavity of female, 8- to 12-week-old C57BL/6 or B cell-deficient μMT C57BL/6 recipients. Secondary grafts were placed in the cervical area of the neck. Survival of heart grafts was assessed by twice weekly palpation of the graft, and rejection was the day the heart graft completely stopped beating. Number of animals per group are indicated in each figure; all mice were randomly co-housed in groups of 2–5/cage.

### Sensitization

2W-OVA F1 mice were sacrificed, their spleens were collected, and processed into a single cell suspension by straining through a 70 µm filter, then resuspended. 4 × 10^6^ cells were injected subcutaneously near each of four limb joints and intraperitoneally, for a total dose of 20 × 10^6^ cells.

### Drug Treatment

Animals were given intraperitoneal CTLA4-Ig (Abatacept, Bristol Myers-Squibb, New York, NY, USA) or isotype Ig control (VEGF-Ig; Avastin, Genentech, South San Francisco, CA, USA) twice a week, with an initial dose of 1 mg and subsequent doses of 0.5 mg. Animals given steroids were given a single 200 µg bolus dose of methylprednisone (Solu-Medrol, Pfizer, New York, NY, USA) on day 6 posttransplantation.

### Flow Cytometry

Splenocytes or heart grafts were collected and prepared for flow cytometry, typically running 10^7^ splenocytes or 1/2 of a heart graft per sample. Dead cells were excluded using AquaFluor LiveDead solution. An additional dump channel was used to exclude irrelevant cells using a mixture of antibodies (DX5, CD11c, F4/80, Gr-1, Ter119, CD19, CD4, or CD8) depending on the stain. Additional antibodies for CD90.2, CD4, CD8, CD44, CD62L, IFNγ were used to stain T cells. 2W(EAWGALANWAVDSA):I-A^b^ tetramer (NIH) and OVA (SIINFEKL):H-2K^b^-PE pentamer (Proimmune, Oxford, UK) or SIINFEKL:H-2K^b^ tetramer (NIH) incubation was performed 30 min at room temperature prior to additional antibodies.

### *In Vivo* Cell Killing Assay

Splenocytes from C57BL/6 mice were collected, their red blood cells lysed, and then counted. Cells were then mixed with CellTrace CFSE (ThermoFisher, Waltham, MA, USA) in PBS at a 15-fold concentration difference between high and low labeled cells. Cells were washed and incubated with either a control peptide [SYIPSAEKI (MBL International Corporation, Woburn, MA, USA); CFSE^hi^ cells] or OVA peptide [SIINFEKL, CFSE^lo^ cells (synthesized by J Collier Lab, Duke University)]. Cells were then mixed and 2 × 10^6^ cells were injected into recipient mice that were naïve, immunized, or immunized + CTLA4-Ig. Recipient animals were sacrificed 3 h later, their spleens harvested, mashed, and run on a flow cytometer. Specific lysis was calculated using the following formula: %Specific Lysis = (1 − (%Sample CFSE^lo^ cells) × (% Naïve CFSE^hi^/% Sample CFSE^hi^ cells)/% Naïve CFSE^lo^ cells) × 100.

### *In Vivo* IFNγ Production Assay

C57BL/6 mice received BALB/c heart transplants, and then, 5 days later, were injected with 1 mg of CTLA4-Ig i.v.; 32 h later, 250 µg brefeldin A was injected intravenously; 15 h after that, recipients were sacrificed and their hearts collected, digested, and mashed through a 70 µm strainer. Staining was performed in an ice-water bath in the presence of brefeldin A to prevent the release of IFNγ, and then stained for flow cytometry as described above. In order to normalize across multiple experiments, in each experiment, the percentage of IFNγ^+^ cells among untreated animals was averaged. Individual values from that experiment were calculated as (%IFNγ^+^/Average% IFNγ^+^ of untreated controls) × 100.

### *In Vitro* Stimulation for IFNγ Staining

Stimulator splenocytes from TCRβ^−/−^ C57BL/6 mice or F1 mice were treated with ACK lysis buffer (Sigma, St. Louis, MO, USA). F1 splenocytes were depleted of T cells with anti-CD90 and two consecutive incubations with rabbit complement at 37°C. 60 × 10^6^ splenocytes of each group were then incubated overnight with 5 µg/mL LPS. The following day, 1 × 10^6^ responder cells were incubated with 5 × 10^5^ stimulators (200 µL per well) in triplicate in a 37°C incubator. 18 h later, 1 µg of Golgi Plug (BD Biosciences, San Jose, CA, USA) was added and incubated an additional 6 h. Extracellular staining was performed in an ice-water bath, cells were fixed, and then stained for intracellular IFNγ.

### Cell Harvest for Flow Cytometry

Spleens were harvested and passed through a 70 µm cell strainer, then, splenocytes lysed in 1 mL ACK lysis buffer (Quality Biological, Gaithersburg, MD, USA) and resuspended in 2% FBS in PBS for cell counting or flow cytometry staining. Prior to harvest, hearts were flushed with HBSS buffer (Thermo Fisher, Waltham, MA, USA) with heparin to minimize blood-derived lymphocytes being included in the graft-infiltrating cell population. Hearts were cut into approximately 2 mm^3^ fragments and placed in HBSS buffer containing collagenase II (Sigma-Aldrich, St. Louis, MO, USA), HEPES (Thermo Fisher, Waltham, MA, USA), and DNAse I (Thermo Fisher, Waltham, MA, USA), and incubated at 37°C for 20 min prior to spinning down and passing through a 70 µM cell strainer, and then used in flow cytometry analysis.

### Histology

Hearts were removed, cut into half, and fixed in 10% formalin for 48 h, and then embedded in paraffin. Sections were cut and stained by Hematoxylin and Eosin. Slides were then scanned using the CRI Pannoramic Whole Slide Scanner (Perkin Elmer, Melville, NY, USA). Grafts were scored in a single blind manner on a 10-point scale, with 0–3 points given for gross histopathological abnormalities, 0–3 points for scarring and decellularization, and 0–4 points for extent of mononuclear cell infiltration.

### DSA Staining

To determine titers of DSA in the serum of recipients, 10^6^ BALB/c splenocytes were incubated for 30 min at 4°C with 5 µL of serum from recipient mice. Cells were then washed and incubated with anti-CD19-APC and anti-IgG-FITC antibodies, and run on a flow cytometer. CD19^−^ cells were gated and the mean fluorescent intensity of anti-IgG-FITC was determined.

### Statistics

Statistical analysis was performed using GraphPad Prism (La Jolla, CA, USA). Graft survival curves significance was assessed using a Mantel-Cox log rank test. Statistically significant differences between two groups were determined by unpaired two-tailed *t*-tests. In comparisons between multiple groups, a one-way ANOVA test was used with a Holm–Sidak correction.

## Results

### Delayed CTLA4-Ig Treatment Rescues Acutely Rejecting Allografts Independently of B Cells

**Figure 1:**
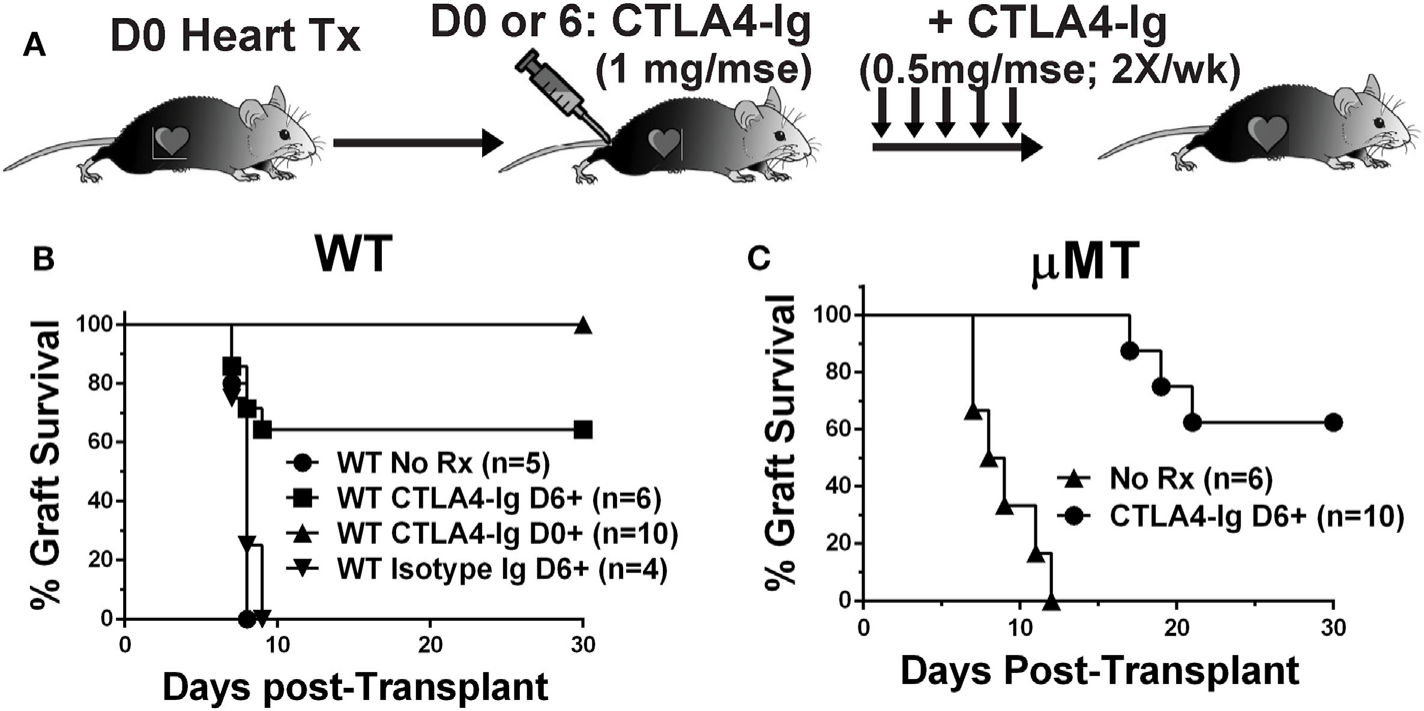
Delayed CTLA4-Ig prevented acute rejection in C57BL/6 WT and μMT recipients. **(A)** Experimental design. **(B)** WT or **(C)** μMT recipients were transplanted with BALB/c donor hearts and received no treatment (No Rx), or CTLA4-Ig given twice a week starting on D0 (CLTA4-Ig D0+) or on D6 (CLTA4-Ig D6+), or isotype Ig starting on D6, for up to 30 days. Graft survival was monitored for 30 days.

To test the hypothesis that CTLA4-Ig can inhibit ongoing and established T cell responses, we compared the effects of CTLA4-Ig, given twice a week from postoperative day 6 posttransplantation of BALB/c hearts, in WT and B cell-deficient (μMT) recipients (Figure 1A). Delayed CTLA4-Ig rescued the heart allografts in approximately 60% of WT recipients, which retained functional hearts at day 28 posttransplantation (Figure 1B). Recipients treated with control human IgG1 fusion protein promptly rejected their allografts (Figure 1B). In the absence of CTLA4-Ig, μMT recipients rejected BALB/c hearts comparably to WT recipients (median survival of 8.5 vs. 8.0 days, respectively; *p* = 0.29). Similar to that observed with WT recipients, delayed CTLA4-Ig treatment beginning on day 6 posttransplantation rescued 60% of BALB/c grafts (Figure 1C). We confirmed that at the time delayed CTLA4-Ig treatment was initiated, there was appreciable alloreactive T cell expansion and differentiation of IFNγ-producing cells (Figure S1 in Supplementary Material). These observations demonstrate that delayed CTLA4-Ig can successfully halt acute T cell-mediated rejection and can do so independently of its ability to inhibit B cells and DSA-production.

### Delayed CTLA4-Ig Did Not Reverse T Cell Graft Infiltration

**Figure 2:**
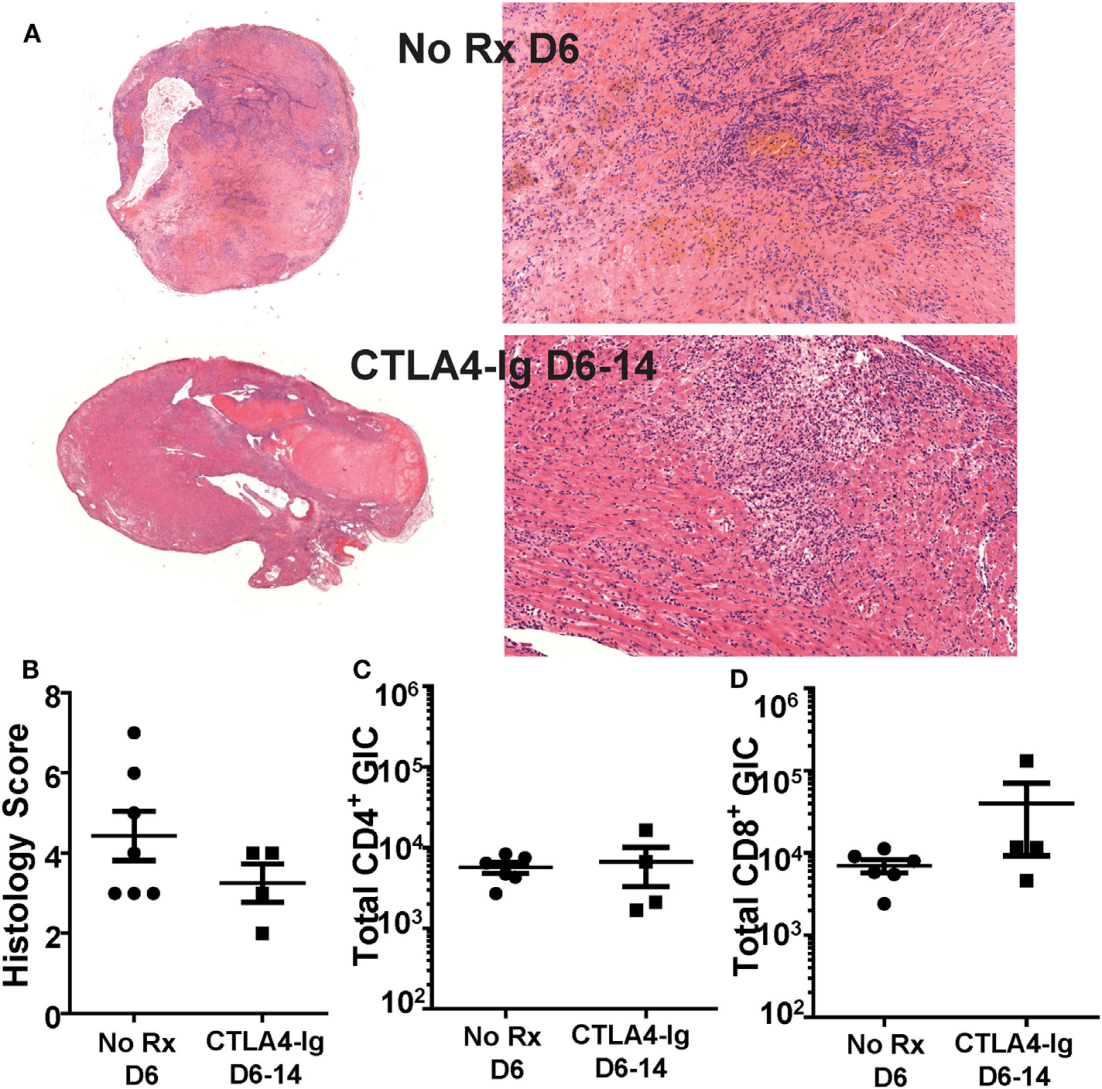
Graft infiltrating T cell numbers were not diminished by delayed CTLA4-Ig treatment. Transplanted BALB/c hearts were harvested on day 6 posttransplantation (No Rx D6) or on D14 from recipients receiving CTLA4-Ig treatment D6, 9, and 13 (CTLA4-Ig D6-14). Animals from each group were sacrificed simultaneously. **(A)** Histology at 1× and 20× from representative No Rx D6 (*N* = 7) and CLTA4-Ig D6-14 (*N* = 4) hearts. **(B)** Heart histology slides were scored for signs of damage and cellular infiltrate. **(C,D)** Total number of heart graft infiltrating CD4^+^ and CD8^+^ T cells (GIC) were enumerated by flow cytometry, and data are presented from individual mice (*N* = 4 or 6 per group) as mean ± SEM.

We next tested whether the ability of delayed CTLA4-Ig to halt ongoing allograft rejection was dependent on inhibiting T cell infiltration into the graft. Because μMT mice may have a potentially aberrant T cell compartment, we focused all subsequent experiments on C57BL/6 recipients of BALB/c heart grafts. Recipients were treated with CTLA4-Ig on days 6, 9, and 13 posttransplantation, and sacrificed on day 14. These grafts were compared to hearts from recipients sacrificed on day 6 posttransplantation, the day CTLA4-Ig treatment commenced. Consistent with the histology of the allografts (Figures 2A,B), we observed no significant change in the total number of infiltrating CD4^+^ or CD8^+^ T cells in grafts on day 6 compared to D6-14 CTLA4-Ig treated hearts (Figures 2C,D). Thus, we conclude that the inhibition of T cell infiltration into the allograft was not the mechanism by which CTLA4-Ig halted ongoing allograft rejection.

### Delayed CTLA4-Ig Inhibited *In Vivo* T Cell Cytolytic Function

**Figure 3:**
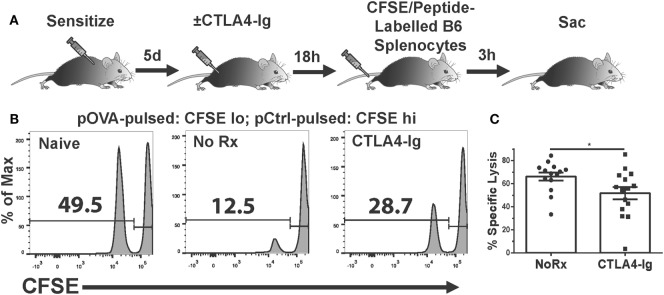
Delayed CTLA4-Ig inhibits antigen-specific CD8^+^ T cell-mediated cytolysis *in vivo*. **(A)** Cartoon depicting the experiment. C57BL/6 mice were immunized with 2W-OVA.F1 splenocytes and treated with CTLA4-Ig on 6 day postimmunization, and 18 h later, received by i.v. injection syngeneic splenocytes pulsed with control peptide (pCtrl; labeled with high CFSE) and syngeneic splenocytes pulsed with OVA peptide [SIINFEKL (pOVA); labeled with low CFSE]. At 3 h after CFSE cell injection, the spleens were harvested and the specific lysis of SIINFEKL^+^ cells compared to control cells from each mouse was enumerated. **(B)** Sample flow plots depicting CFSE-labeled cell recovery from naïve, untreated, and CTLA4-Ig treated animals are shown. **(C)** Specific lysis of SIINFEKL^+^ cells in immunized animals, *N* = 14 or 15 per group from three experiments. Data are presented from individual mice and as mean ± SEM; *p < 0.05 by two-tailed *t*-test.

The absence of a significant effect on T cell graft infiltration prompted the hypothesis that CTLA4-Ig inhibited T cell function. While T cells can be primed by the direct, semi-direct, and indirect pathways (29–34), only direct recognition by recipient CD8^+^ T cells of intact MHC Class I on allogeneic target cells results in their lysis and acute allograft rejection (35–38). Therefore, we investigated whether the *in vivo* cytolytic activity of alloreactive CD8^+^ T cells was reduced by CTLA4-Ig. To avoid the contribution of DSA- and NK-mediated cell lysis, we developed an experimental system in which C57BL/6 mice were immunized with OVA.BALB/c × C57BL/6 (F1) splenocytes to generate cytotoxic CD8^+^ T cells specific for the OVA peptide (SIINFEKL) presented on H-2K^b^. On day 6 postimmunization, when significant CTLs had been generated (21), we administered CTLA4-Ig and measured *in vivo* pOVA-specific killing 24 h later. Specifically, a mixture of syngeneic CFSE^lo^ cells loaded with SIINFEKL peptide, and CFSE^hi^ cells presenting an irrelevant peptide, were injected intravenously at a 1:1 ratio (Figure 3A). These syngeneic targets should avoid detection by OVA-specific and allo-specific antibodies, NK cells, and other innate cells capable of allo-recognition, while the 24 h wait allowed for the steady-state distribution of CTLA4-Ig. Three hours after the infusion of CFSE-labeled target cells, the spleens were harvested from untreated or CTLA4-Ig-treated mice and the numbers of CFSE-labeled cells were quantified. SIINFEKL-pulsed cells were normalized to control peptide-pulsed cells to determine OVA:K^b^-specific lysis in control and CTLA4-Ig-treated mice. We observed a modest, but statistically significant, decrease in the specific lysis of SIINFEKL-pulsed cells in the CTLA4-Ig treated animals compared to untreated controls (51.7 vs. 66.2%; *p* = 0.04; Figures 3B,C).

### Delayed CTLA4-Ig Inhibited *In Vivo* IFNγ Production

**Figure 4:**
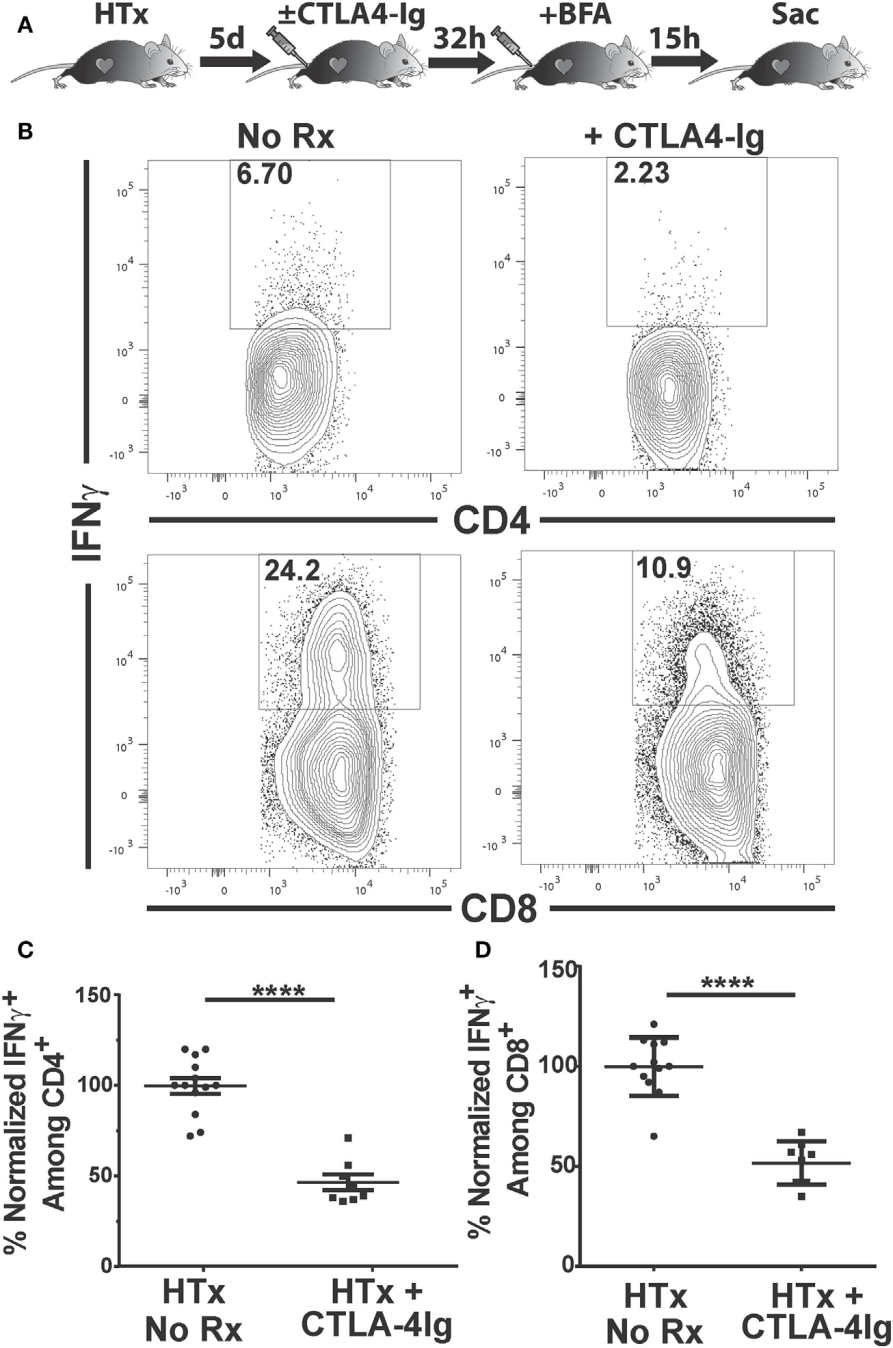
Delayed CTLA4-Ig blocks the *in vivo* production of IFNγ by graft-infiltrating T cells. C57BL/6 mice were transplanted with BALB/c hearts and either remained untreated or given CLTA4-Ig 5 day posttransplantation. On day 6, recipients were injected with brefeldin A and 5 h later, mice were sacrificed, and IFNγ production by the graft-infiltrating T cells was determined by intracellular staining. **(A)** Cartoon depicting the experiment. **(B)** Sample IFNγ flow cytometry patterns from untreated (left column) or CLTA4-Ig treated (right column) heart transplant recipients. **(C,D)** Percentage of IFNγ-producing, **(C)** CD4^+^, and **(D)** CD8^+^ graft-infiltrating T cells in CTLA4-Ig treated recipients (HTx + CTLA4-Ig) was normalized and compared with the untreated group (HTx + No Rx). Data are presented from individual mice, *N* = 8 or 12 per group from at least three experiments, as mean ± SEM; ****p < 0.0001 by two-tailed *t*-test.

IFNγ is a pro-inflammatory cytokine produced by CD4^+^ and CD8^+^ T cells that has been associated with and is predictive of acute allograft rejection (39, 40). The production of IFNγ by graft-infiltrating recipient CD4^+^ T cells recognizing MHC class II-expressing cardiac allografts, together with the expression of IFNγR on donor grafts, has been shown to be necessary for the efferent phase of cardiac allograft rejection (41). We tested whether delayed CTLA4-Ig treatment could inhibit T cell IFNγ production *in vivo*. IFNγ production is tightly regulated to prevent immunopathology, undergoes rapid on/off cycling in response to antigen, and is quickly secreted upon production (42, 43). Thus, we used a previously described approach to enumerate T cells that are actively synthesizing IFNγ *in vivo* that circumvents the need for *ex vivo* Ag stimulation (44, 45). Recipients of BALB/c hearts received CTLA4-Ig on day 5 posttransplantation, and brefeldin A 32 h later. Brefeldin A prevents cytokine secretion and thus allows its accumulation in the T cells actively producing IFNγ *in vivo*. At 15 h post-brefeldin A injection, the heart grafts and spleens were harvested, graft-infiltrating and spleen cells were isolated in media containing brefeldin A, and the frequency of T cells producing IFNγ were enumerated (Figure 4A). Approximately 6% of CD4^+^ and 20% of CD8^+^ T cells within the graft were producing IFNγ (Figures 4B–D); in contrast ≤0.1% of CD4^+^ and CD8^+^ T cells in the spleen were IFNγ^+^ (Figure S2 in Supplementary Material), consistent with the notion that *in vivo* IFNγ production is tied to contemporaneous antigenic stimulation and is facilitated by high intragraft antigen concentration relative to the spleen. CTLA4-Ig treatment induced a 50% decrease in the percentage of IFNγ^+^-producing CD4^+^ and CD8^+^ T cells in the graft, compared to untreated controls (Figures 4B–D), thus underscoring the continued requirement of CD28:CD80/86 interactions for stimulating T cell effector function *in vivo*.

### Delayed CTLA4-Ig Modestly Reduced the Total Numbers of Donor-Specific IFNγ-Producing T Cell Precursor

**Figure 5:**
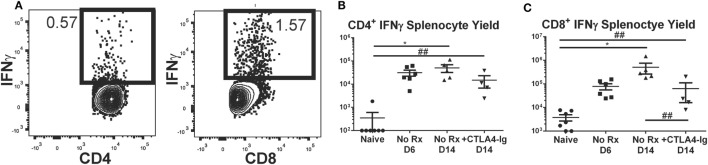
Effect of delayed CTLA4-Ig on the precursor frequency of IFNγ-producing T cells in fully allogeneic heart transplant recipients. BALB/c hearts were transplanted into C57BL/6 mice and were not treated (No Rx) or were treated with CLTA4-Ig on D6, 9, and 13 post-Tx (CTLA D6-14), and sacrificed on day 14 posttransplantation and restimulated *ex vivo*. **(A)** Sample flow plots of allospecific IFNγ staining of T cells from No Rx recipients. **(B,C)** Total number of allospecific IFNγ-producing CD4^+^ **(C)** or CD8^+^ **(D)** from the spleens of recipients receiving WT BALB/c hearts. Data shown from individual mice (*N* = 4–7/group) and as mean ± SEM. *p < 0.05, by one-way ANOVA. ^##^*p* < 0.01 by two-tailed *t*-test.

It has been reported that about 90% of the antigen-specific effector T cells present at the peak of a primary response die during the contraction phase, leaving a residual population of long-lived memory T cells (46, 47). Because CTLA4-Ig has been previously shown to be ineffective at controlling memory T cells, we next tested whether the ability of delayed CTLA4-Ig to maintain long-term graft acceptance was due to the reduced numbers of alloreactive IFNγ-producing T cell precursors. The total number of these IFNγ-producing T cell precursors was determined following *ex vivo* stimulation with donor- vs. recipient-matched cells (Figure 5A), from recipients of BALB/c hearts treated with delayed CTLA4-Ig for 8 days (from days 6 to 14 posttransplantation) compared to untreated transplant recipients sacrificed on days 6 or 14 posttransplantation, and also to non-transplanted naïve controls. On day 6 posttransplantation, there was already a significant increase in the total number of alloreactive IFNγ-producing CD4^+^ T cell precursors, which remained at comparable levels on day 14 (Figure 5B). Delayed CTLA4-Ig treatment did not significantly reduce the total number of alloreactive IFNγ-producing CD4^+^ T cell precursors compared to untreated day 6 or 14 controls, although we noted a downward trend. The total number of alloreactive IFNγ-producing CD8^+^ T cell precursors peaked on day 14 (Figure 5C), and delayed CTLA4-Ig significantly blunted the day 6–14 increase. Nevertheless, the total number of alloreactive IFNγ-producing CD8^+^ T cells in CTLA4-Ig-treated recipients on day 14 posttransplantation was increased compared to naïve controls, consistent with the notion that CTLA4-Ig was indeed able to control these donor-specific IFNγ-producing T cell precursors and to maintain graft acceptance after a rejection crisis.

### Delayed CTLA4-Ig Reduced the Frequency of Graft-Specific CD4^+^ but Not CD8^+^ T Cells

**Figure 6:**
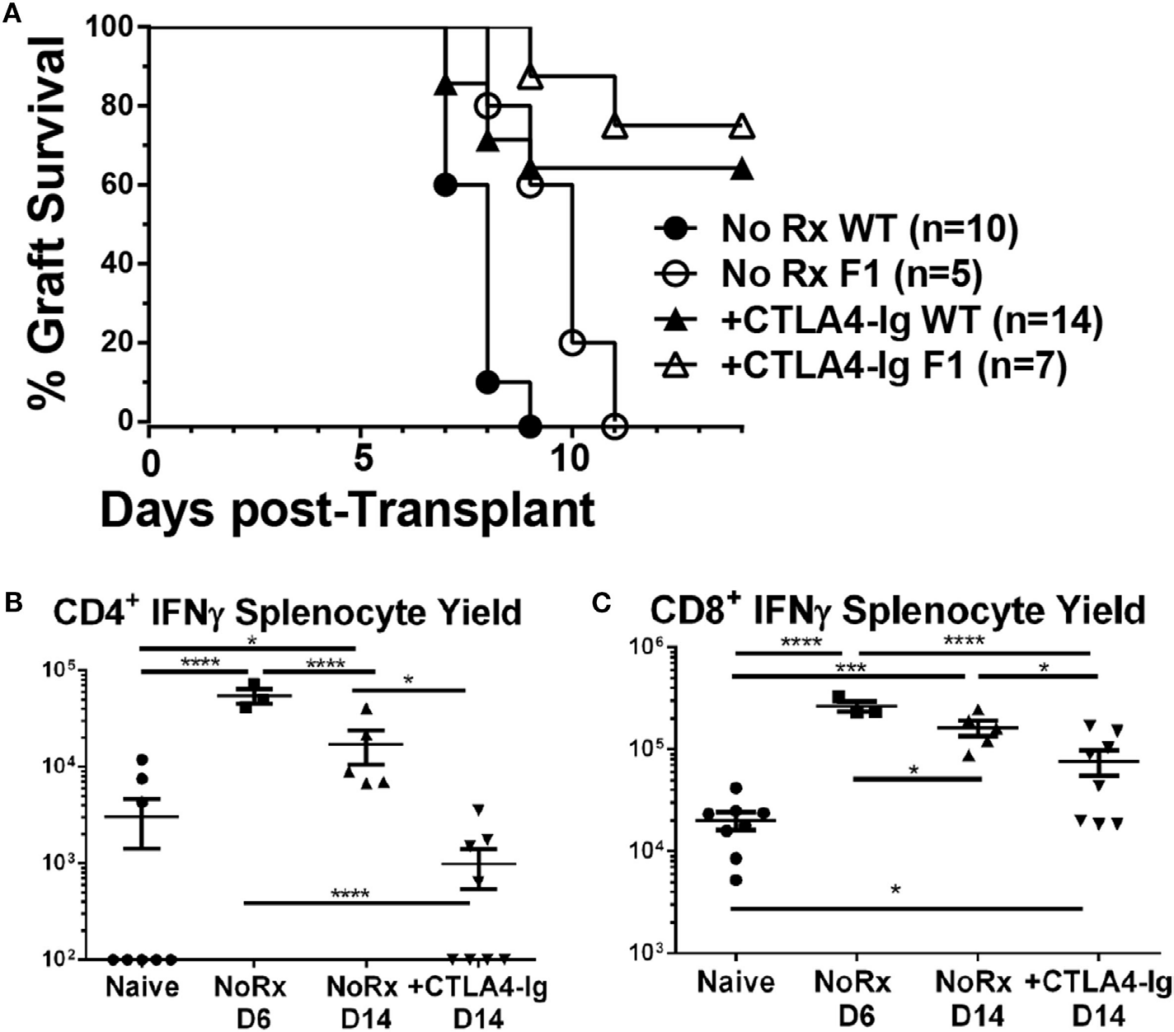
Effect of delayed CTLA4-Ig on the precursor frequency of IFNγ-producing T cells in haplo-mismatched F1 heart transplant recipients. 2W-OVA.F1 (BALB/c X 2W-OVA.C57BL/6) hearts were transplanted into C57BL/6 mice as described for Figure 5. **(A)** Comparison of heart survival, **(B,C)** total number of allospecific IFNγ-producing CD4^+^ **(B)** or CD8^+^ **(C)** T cells from the spleens of recipients receiving F1 hearts. Data shown from individual mice (*N* = 3–8/group) as mean ± SEM. *p < 0.05, ***p < 0.001, ****p < 0.0001 by one-way ANOVA.

Since alterations in the frequency of IFNγ-producing T cells could be the result of suppression, deletion, or immune skewing to T cells producing other cytokines, we additionally tracked the fate of endogenous graft-reactive T cells in the haplo-mismatched model where BALB/c × C57BL/6 F1 hearts expressing the 2W-OVA transgene (2W-OVA.F1) were transplanted into C57BL/6 recipients. 2W-OVA.F1 grafts were rejected 2 days slower than fully mismatched BALB/c grafts [median graft survival of 8.0 vs. 10.0 days; *p* = 0.01 (Figure 6A)]. Thus, subtle differences in the kinetics, compared to a full-mismatched BALB/c allograft, of alloreactive IFNγ-producing CD4^+^ and CD8 T^+^ cell accumulation were observed with the B/c compared to the 2W-OVA.F1 heart grafts (Figures 5B,C vs. Figure 6). IFNγ-producing CD4^+^ T cell numbers peaked on day 6 in recipients receiving 2W-OVA.F1 heart grafts and then declined by day 14, and delayed CTLA4-Ig promoted the contraction of these cells (Figure 6B), potentially by inhibiting further proliferation or enhancing cell death. In addition, the IFNγ^+^CD8^+^ T cell frequencies were maintained from day 6–14, and delayed CTLA4-Ig also promoted the contraction of these cells (Figure 6C).

**Figure 7:**
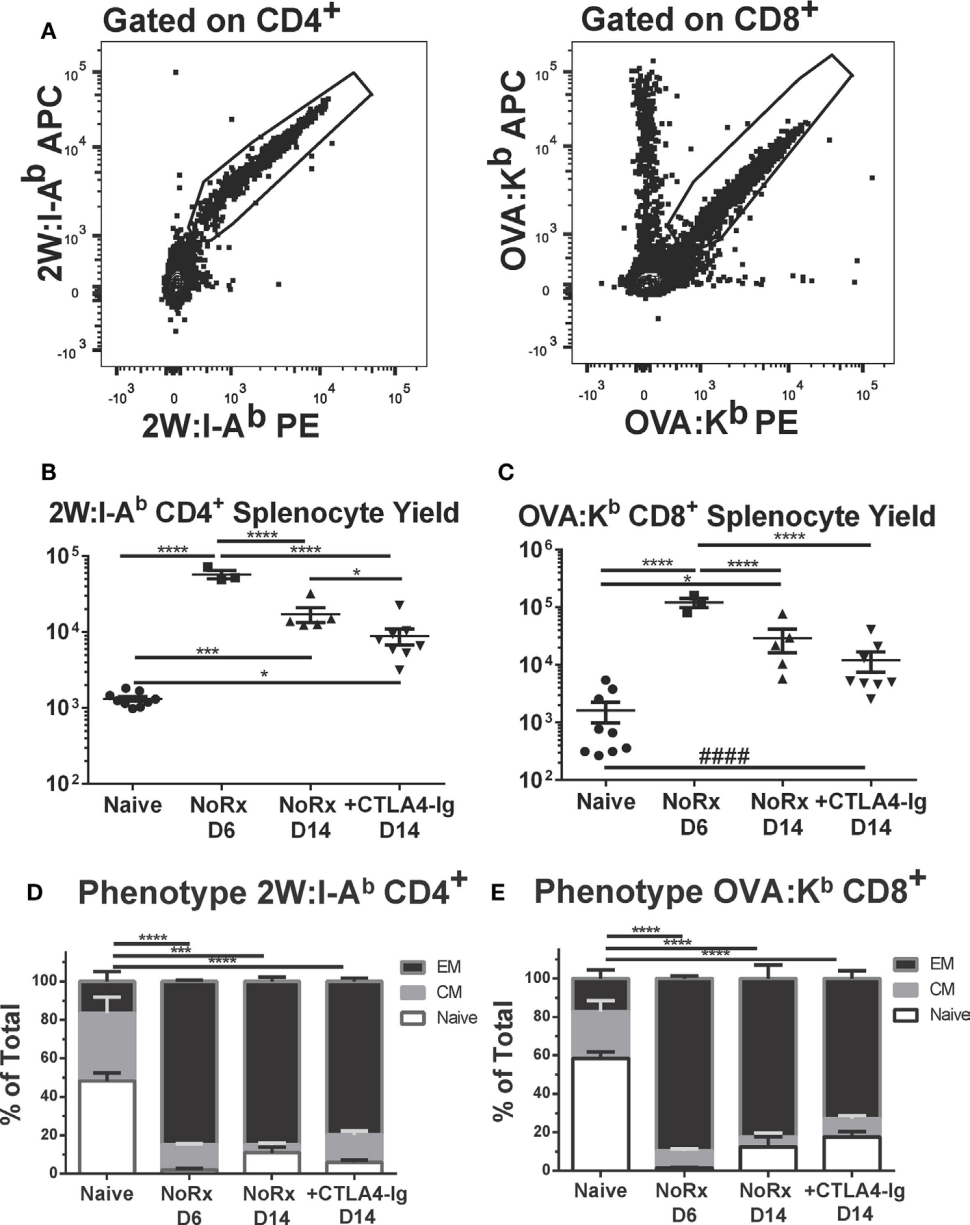
Delayed CTLA4-Ig moderately reduced the frequency of endogenous graft-specific T cells. **(A)** Sample gating strategies for the identification of 2W:I-A^b^-specific CD4^+^ T cells or OVA-H-2K^b^-specific CD8^+^ T cells. **(B,C)** C57BL/6 mice were transplanted with 2W-OVA.F1 hearts, without treatment (AR) or were treated with CLTA4-Ig on D6, 9, and 13 post-Tx (CTLA D6-14) prior to sacrifice on post-transplant day 14. Recipient spleens were analyzed for the total number of **(B)** 2W:I-A^b^-specific CD4^+^ T cells; **(C)** OVA:H-2K^b^-specific CD8^+^ T cells. **(D,E)** The percentage of CD44^+^CD62L^−^ effector memory (EM), CD44^+^CD62L^+^ central memory (CM), and CD44^−^CD62L^+^ (Naïve) within the **(D)** 2W:I-A^b^-specific CD4^+^ T cells or **(E)** OVA-H-2K^b^-specific CD8^+^ T cells, with comparison to Naïve. Data shown from individual mice (*N* = 3–9/group) as mean ± SEM. *p < 0.05, ***p < 0.001, ****p < 0.0001 by one-way ANOVA. ^####^*p* < 0.0001 by two-tailed *t*-test.

Using this model, we have previously reported that the endogenous graft-reactive CD4^+^ and CD8^+^ T cells could be enumerated using 2W:I-A^b^ and OVA:K^b^ tetramers, respectively (21). Here, we demonstrate that the numbers of 2W:I-A^b^ CD4^+^ and OVA:K^b^ CD8^+^ T cells expanded significantly from their baseline population and peaked on day 6, and that the contraction of CD4^+^ T but not CD8^+^ T cells on day 14 posttransplantation was modestly enhanced by delayed CTLA4-Ig (Figures 7A–C). Significantly higher percentages of 2W:I-A^b^ CD4^+^ and OVA:K^b^ CD8^+^ T cells were of the CD44^+^CD62L^−^ T effector memory (EM) phenotype in the untreated recipients on days 6 and 14 posttransplantation, compared to naïve controls (Figures 7D,E). Additionally, the majority of 2W:I-A^b^ CD4^+^ and OVA:K^b^ CD8^+^ T cells displayed a CD44^+^CD62L^−^ T EM phenotype in the delayed CTLA4-Ig treated mice and were comparable to the frequencies observed in untreated recipients. Collectively, these observations suggest that successful modulation of acute rejection and ensuing graft survival upon delayed CTLA4-Ig treatment was mediated, at least in part, by the ability of CTLA4-Ig to control endogenous alloreactive effector T cells, including alloreactive T cells with EM phenotype.

### CTLA4-Ig Treatment Promoted Long-term Acceptance of Second, Donor-Specific Heart Allografts in Recipients That Were Rescued from Acute Rejection

**Figure 8:**
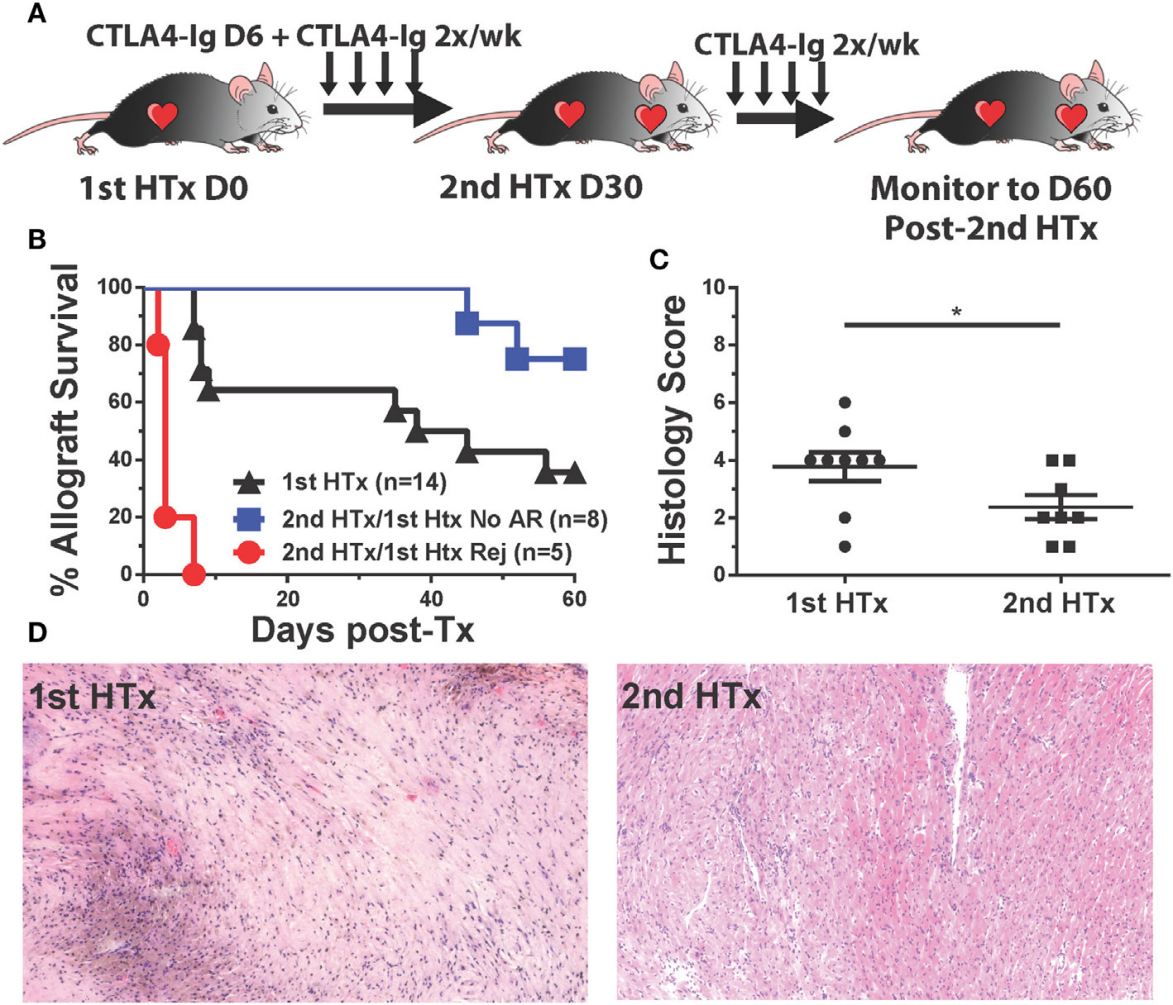
CTLA4-Ig treatment promotes long-term acceptance of second, donor-specific heart allografts in recipients that were successfully treated for acute rejection. C57BL/6 recipients of primary BALB/c heart transplant were treated with CTLA4-Ig 2×/week from day 6 posttransplantation. At 30 days post primary transplant, a second heart was transplanted into each recipient, and the survival of the secondary hearts was monitored. **(A)** Cartoon depicting the experimental protocol. **(B)** Graft survival curve for primary (*N* = 14) and secondary heart grafts (*N* = 5 and *N* = 8). Survival is determined from the time each allograft was transplanted. **(C)** Histological scoring of surviving heart grafts at sacrifice. **(D)** Sample histological slides from primary and secondary heart grafts (20×). Data shown from individual mice (*N* = 8–9) as mean ± SEM. *p < 0.05 by two-tailed *t*-test.

The initiation of treatment with CTLA4-Ig on day 6 posttransplantation resulted in two groups of recipients: those that fully rejected their grafts between days 9 and 15 posttransplantation, and those with surviving heart grafts past day 30 posttransplantation, although these grafts were beating weakly relative to grafts where CTLA4-Ig was initiated at the time of transplantation (Figure 1A). We hypothesized that the sustained but poorer graft function could be explained by either initial damage sustained in the first 6 days posttransplantation, or to chronic damage due to incompletely controlled alloreactivity. To distinguish between these possibilities, we transplanted a second BALB/c heart into recipients with surviving hearts on day 30 posttransplantation of the first heart and maintained them on twice a week CTLA4-Ig (Figure 8A). Seventy-five percent of these secondary grafts survived up to 60 days posttransplantation (Figure 8B), and when the histology of these secondary grafts was examined at 60 days post-secondary transplant and compared to surviving primary grafts (also at day 60 posttransplantation of the first heart), we observed significantly less damage to the heart tissues and less graft infiltration in secondary grafts (Figures 8C,D). These observations are consistent with the conclusion that continued CTLA4-Ig was able to substantially control memory T cell-mediated rejection, although not as effectively as controlling T cell responses in naïve recipients. Noteworthy was the observation that in the recipients wherein delayed and continuous CTLA4-Ig treatment failed to save the first allograft, a donor-matched allograft transplanted on day 30 post-first transplantation was rejected in an accelerated fashion, despite the absence of circulating DSA [Figure 8B; Figure S3D in Supplementary Material (21)]. We observed that animals that rejected their grafts and were sacrificed on day 14 tended to be the highest producers of IFNγ, although this trend did not reach statistical significance (data not shown).

## Discussion

Observations from experimental models of heterologous memory (24, 48) together with clinical observations of belatacept-resistant acute rejection (8–10, 49) support a hypothesis that memory T cells do not require CD28–CD80/86 interactions for their re-stimulation, or that there is redundancy of activating costimulatory molecules. However, this hypothesis appears to be at odds with the clinical observations that despite the occurrence of acute rejection in patients on belatacept [9.1–14.2% biopsy-proven acute rejection at 36 months posttransplantation (8, 9)], once acute rejection is successfully treated with bolus steroids, the grafts go on to survive at rates that are comparable to conventional cyclosporine-based immunosuppression [4.7–5.4% graft loss for belatacept vs. 9.8% for cyclosporine at 84 months posttransplantation (10)]. Even more impressive is the observation that the eGFR continue to improve at a rate of 1.30 ml per minute per 1.73 m^2^ per year under belatacept (10). In a smaller study (*N* = 20) by Kirk et al. (50) where 5 of 10 patients were successfully weaned to belatacept monotherapy, but where five failed weaning and experienced subclinical (*N* = 2) or acute rejection (*N* = 3), two patients were subsequently re-weaned to belatacept monotherapy. These and other observations of successful maintenance of long-term graft survival with belatacept-based immunosuppression after rejection (51–53) suggest a need for a closer examination of the conditions that permit belatacept efficacy despite acute rejection, and those that result in belatacept-resistant rejection even in non-high risk recipients.

In this study, we used a model of HLA and minor antigen-mismatched, as well as a haplo-mismatched, heart transplantation in mice to investigate the impact of delayed CTLA4-Ig on endogenous polyclonal donor-reactive T cells and ongoing acute rejection. CTLA4-Ig treatment was initiated on day 6 posttransplantation, when there was already significant if not peak levels of primed CD4^+^ and CD8^+^ T cells, and substantial T cell infiltration into the allograft. In the absence of intervention, the allogeneic hearts completely stopped beating on day 8 posttransplantation, whereas CTLA4-Ig was able to stop the rejection process and promote graft survival in approximately 60% of the recipients. Importantly, delayed CTLA4-Ig was also able to promote graft survival in B cell-deficient recipients, confirming that its efficacy is not limited to its ability to inhibit established B cells and DSA responses (20, 21). We acknowledge a number of caveats of the study. CTLA4-Ig treatment initiated as late as day 6 posttransplantation is not likely to represent a meaningful intervention in the clinic. Additionally, the dose of CTLA4-Ig is higher than used in the clinic (54–56) and by other investigators in mice (57, 58). Belatacept is a 10-fold high-affinity mutant of CTLA4-Ig (59), but does not block mouse B7, justifies the use of higher doses of abatacept, which does bind mouse B7, in mice. Moreover, the half-life of human CTLA4-Ig (abatacept) in mice is likely to be different from belatacept in humans, making exactly matched dosing challenging. Therefore, this study represents a mechanistic test of the efficacy of CTLA4-Ig at inhibiting established ongoing effector T cell responses, at doses that approach maximal efficacy in mice.

We observed that delayed CTLA4-Ig did not reverse T cell infiltration into the graft, but was able to blunt *in vivo* CD8^+^ T cell cytolytic function, as well as IFNγ production by CD4^+^ and CD8^+^ graft-infiltrating T cells under the same conditions that successfully halted the acute rejection process. To our knowledge, the ability of CTLA4-Ig to inhibit *in vivo* cytolytic activity and IFNγ production by primed alloreactive T cells has not been reported in such a transplant setting. Because CD8^+^ T cells are critical mediators of acute rejection that, upon reencounter with cognate antigen, release cytotoxic molecules that cause the lysis of target cells and acute rejection (32, 35, 37), these observations expand our mechanistic understanding of the efficacy of CTLA4-Ig and the continued role of CD28 in regulating these effector T cell functions. Furthermore, we noted that IFNγ-producing T cells were highly enriched in the allograft compared to the spleen, likely reflecting the higher frequency of alloreactive T cells and, potentially, the amount of cognate antigen within the allograft. That CTLA4-Ig was able to reduce the frequency of IFNγ-producing T cells underscores its bioavailability within the allograft and also its potency, especially in light of recent reports indicating that signaling from a single TCR is sufficient to trigger T cell cytokine production and T cell proliferation at a single cell level (60, 61). Finally, our observations can also be placed in the context of the reports (62, 63) showing that the provision of CD28 co-stimulation together with chimeric antigen receptors resulted in enhanced cytolytic activity and IFNγ production by CD8^+^ T cells.

In this study, we also examined the longer-term impact of CTLA4-Ig on the frequency of alloreactive T cells by quantifying the frequency of *ex vivo* allo-stimulated IFNγ-producing cells, as well as by tracking graft-reactive T cells (2W:I-A^b^ and OVA:K^b^) that recognize donor peptides indirectly presented on recipient MHC. With both approaches, we observed that the CD4^+^ and CD8^+^ alloreactive T response peaked, or was substantially expanded by day 6 posttransplantation, and that 7–24 days of treatment with CTLA4-Ig promoted the reduction in alloreactive T cell numbers. We speculate that the contraction phase of the T cell response observed in the spleen (and potentially in the lymph nodes) even in the absence of immununosuppression is not reflected in the graft undergoing acute rejection and may explain the inability of delayed CTLA4-Ig to reduce the number of T cell infiltrating into the graft.

We noted that the alloreactive T cells that remained in delayed CTLA4-Ig treated recipients were enriched for cells with a CD44^+^ memory phenotype; and that CTLA4-Ig was nevertheless able to prolong survival of the primary graft, and also of secondary, donor-matched allografts. These observations underscore the ability of continued CTLA4-Ig treatment to substantially blunt T cell effector function and control memory T cells generated during allograft rejection, and potentially explain the clinical observation that belatacept-based immunosuppression can maintain long-term graft survival after acute rejection has been resolved with bolus steroids therapy. Indeed, we observed that delayed CTLA4-Ig alone had similar efficacy as CTLA4-Ig in combination with a single bolus dose with methylprednisone, given on day 6 posttransplantation, in modulating the frequency of alloreactive T cells and in promoting long-term allograft acceptance (Figure S3 in Supplementary Material). These observations support the conclusion that CTLA4-Ig can indeed control endogenous effector and memory T cells.

We additionally observed that in approximately 40% of recipients, delayed CTLA4-Ig failed to halt the rejection process and the grafts went on to completely stop beating within 4 days of CTLA4-Ig treatment. This failure to rescue from acute rejection could be due to the grafts being too badly injured to recover even if CTLA4-Ig treatment had successfully controlled the alloimmune response comparably to recipients where rejection was halted and the heart grafts continued to beat. Alternatively, delayed CTLA4-Ig treatment did not fully curtail the effector and/or memory T cell response. The rapid rejection of secondary donor-matched heart grafts by these recipients, even when no circulating DSA was detected, suggests that CTLA4-Ig was not able to curtail the memory T cell responses. These observations are reminiscent of clinical observations of belatacept-resistant acute T cell-mediated rejection (8–10), and we are currently investigating whether this model can be utilized for the identification of critical biomarker differences that predict successful avoidance of CTLA4-Ig-resistant acute rejection.

In summary, our studies demonstrate an unexpected efficacy of CTLA4-Ig at stopping established T cell-mediated acute rejection, complementing our previous observations that CTLA4-Ig was able to control established B cell responses (18–21). Because continued CTLA4-Ig therapy was unable to eliminate or reprogram memory alloreactive T cells generated during the acute rejection back to a naïve repertoire, we conclude that its efficacy in treating established rejection is likely due, at least in part, to its ability to blunt *in vivo* cytolytic activity of donor-reactive CD8^+^ T cells, and IFNγ production by graft infiltrating CD4^+^ and CD8^+^ T cells. These observations complement and extend our recent findings of the ability of CTLA4-Ig to blunt rejection in recipients that had been sensitized with donor spleen cells 14 months or more prior to heart transplantation (64). Finally, despite the effects of high dose CTLA4-Ig on T and B, and potentially also on NK cells, dendritic cells, and other innate cells (65, 66), our studies reveal the limits of CTLA4-Ig efficacy, even when used at supra high doses, at controlling effector T cells. These observations pave the way for future studies aimed at defining the critical features of an alloimmune response that defies CTLA4-Ig immunosuppression.

## Ethics Statement

This study was carried out in accordance with the recommendations of the guidelines set forth by the National Institutes of Health and Office of Laboratory Animal Welfare. The protocol was approved by the University of Chicago Insitutional Animal Care and Use Committee.

## Author Contributions

JYoung performed the experiments, analyzed the data, and wrote the manuscript. SHK, JYang and AV performed experiments, analyzed data, and edited the manuscript. DY performed all transplants and assisted with experiments. RS and M-LA provided key scientific insight. AC designed the study, and co-wrote the manuscript.

## Conflict of Interest Statement

The authors declare that the research was conducted in the absence of any commercial or financial relationships that could be construed as a potential conflict of interest.

## Abbreviations

2W, peptide, EAWGALANWAVDSA, or 2W:I-A^b^ tetramers; APC, allophycocyanin; CTLA4, cytotoxic T-lymphocyte-associated protein 4; DSA, donor-specific antibodies; F1, C57BL/6xBALB/c; OVA, ovalbumin or SIINFEKL:H-2K^b^ (OVA) pentamers or tetramers; PE, phycoerythrin; TCR, T cell receptor.
