# Supplementary figures and images for: Successful Treatment of T Cell-Mediated Acute Rejection with Delayed CTLA4-Ig in Mice
Source: Front Immunol. 2017 Sep 20;8:1169. doi: 10.3389/fimmu.2017.01169 (PMC5609110; doi:10.3389/fimmu.2017.01169)

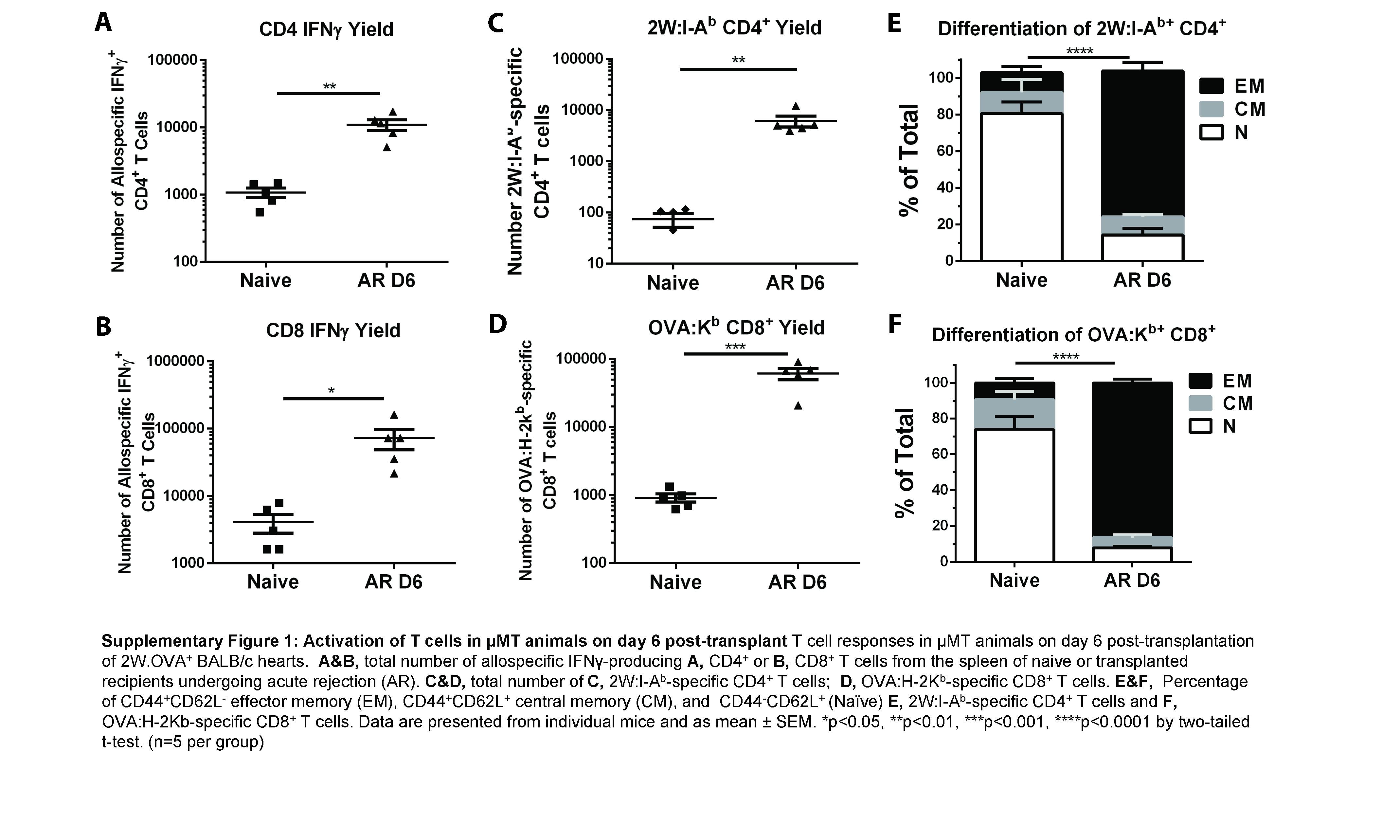

Supplement: Supplementary file 1 [file Image_1.TIF]

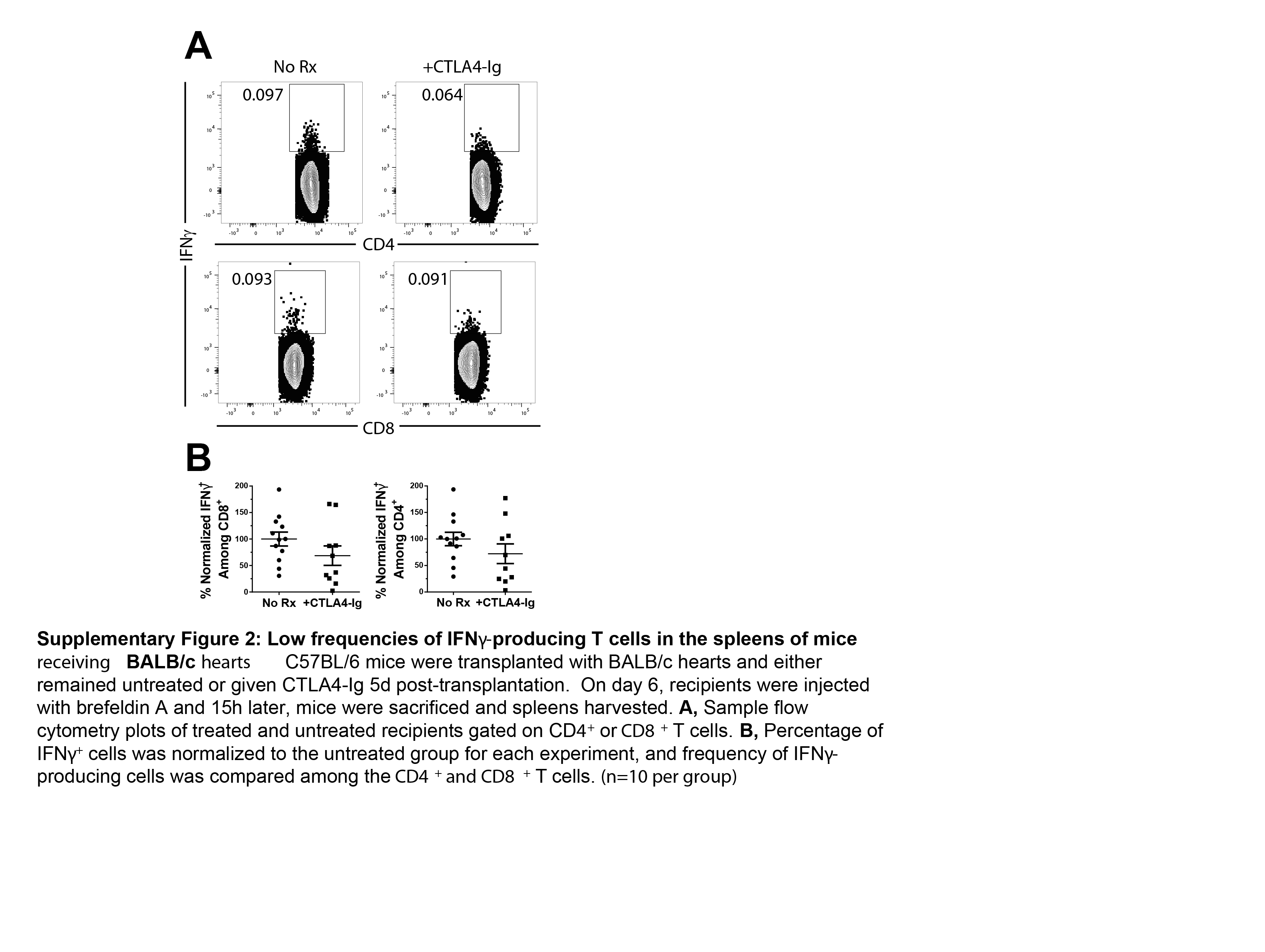

Supplement: Supplementary file 2 [file Image_2.TIF]

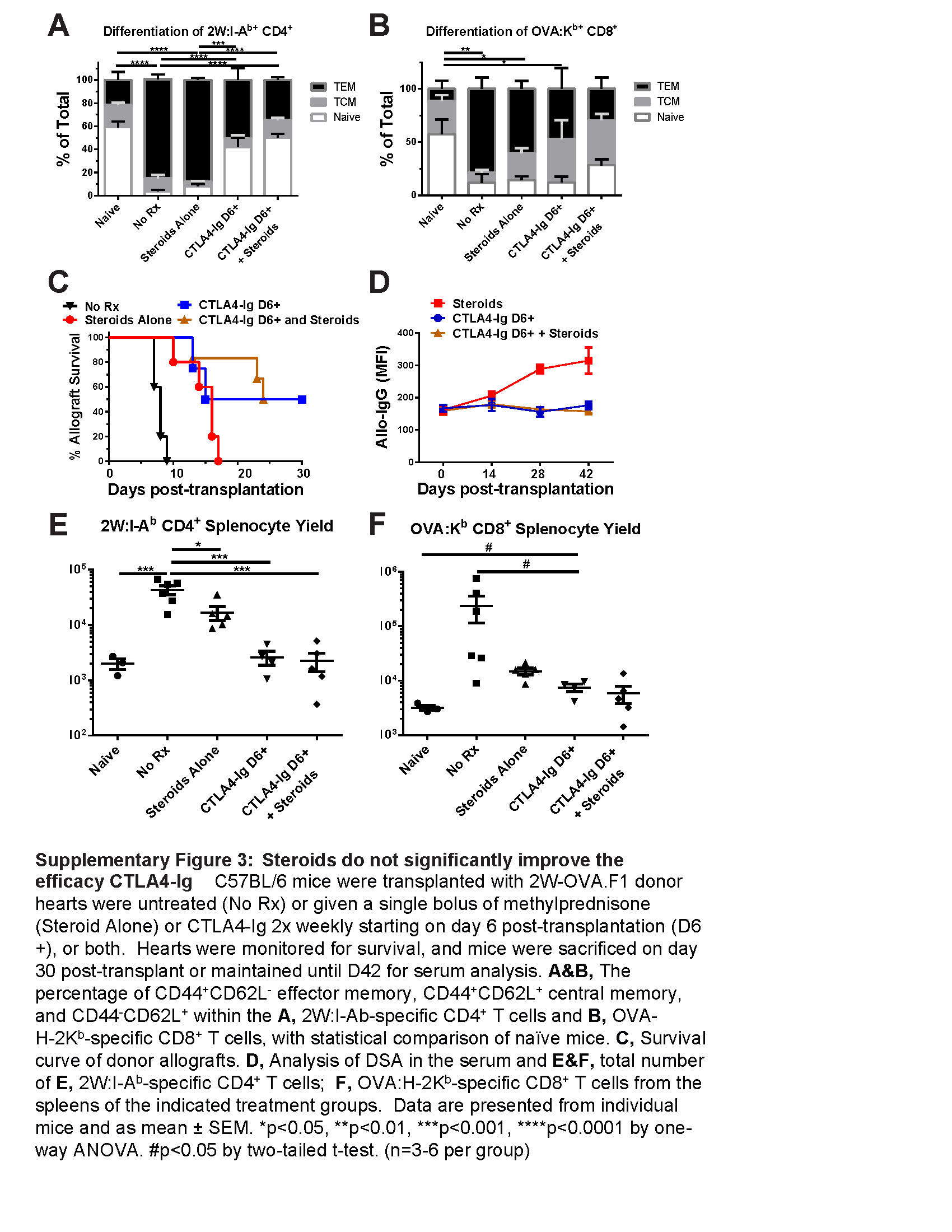

Supplement: Supplementary file 3 [file Image_3.TIF]
